# Differential CLE peptide perception by plant receptors implicated from structural and functional analyses of TDIF-TDR interactions

**DOI:** 10.1371/journal.pone.0175317

**Published:** 2017-04-06

**Authors:** Zhijie Li, Sayan Chakraborty, Guozhou Xu

**Affiliations:** Department of Molecular and Structural Biochemistry, North Carolina State University, Raleigh, North Carolina, United States of America; University of Queensland, AUSTRALIA

## Abstract

Tracheary Element Differentiation Inhibitory Factor (TDIF) belongs to the family of post-translationally modified CLE (CLAVATA3/embryo surrounding region (ESR)-related) peptide hormones that control root growth and define the delicate balance between stem cell proliferation and differentiation in SAM (shoot apical meristem) or RAM (root apical meristem). In *Arabidopsis*, Tracheary Element Differentiation Inhibitory Factor Receptor (TDR) and its ligand TDIF signaling pathway is involved in the regulation of procambial cell proliferation and inhibiting its differentiation into xylem cells. Here we present the crystal structures of the extracellular domains (ECD) of TDR alone and in complex with its ligand TDIF resolved at 2.65 Ǻ and 2.75 Ǻ respectively. These structures provide insights about the ligand perception and specific interactions between the CLE peptides and their cognate receptors. Our *in vitro* biochemical studies indicate that the interactions between the ligands and the receptors at the C-terminal anchoring site provide conserved binding. While the binding interactions occurring at the N-terminal anchoring site dictate differential binding specificities between different ligands and receptors. Our studies will open different unknown avenues of TDR-TDIF signaling pathways that will enhance our knowledge in this field highlighting the receptor ligand interaction, receptor activation, signaling network, modes of action and will serve as a structure function relationship model between the ligand and the receptor for various similar leucine-rich repeat receptor-like kinases (LRR-RLKs).

## Introduction

Since the discovery of the first plant peptide hormone systemin in 1991 [1], secreted small peptides have been recognized as essential mediators in intercellular communication that governs plant growth, development, interaction with environment, and other biological responses [2]. Bioinformatics analyses have predicted more than 1000 putative secretory peptides in *Arabidopsis* genomes [3], many of which have been experimentally confirmed to mediate intracellular communication during a large variety of plant biological processes, such as stem cell homeostasis, cell proliferation, wound healing, hormone sensing, immune defense, and legume symbiosis [4]. One of the best-characterized families of plant peptide hormones is CLE (CLAVATA3 (CLV3)/embryo surrounding region (ESR)-related). In *Arabidopsis thaliana* the CLE family peptides are encoded by 32 genes including CLV3, which express small proteins between 60–120 amino acids long [5–8]. In addition to the N-terminal signal peptide (SP) domain, the CLE proteins contain a large portion of amino acid sequences in the middle known as variable region that have no conservation within the family. However, the C-terminal 12–13 amino acid residues, known as CLE domain, are highly conserved in each member. The middle non-conserved region is mostly dispensable for their in vivo function [9], while chemically synthesized 12–13 amino acid residue peptides corresponding to the CLE domain are fully functional when applied to plants [10–12]. The secreted CLE proteins are post-translationally modified and proteolytically processed to peptides of 12–13 residues with two conserved hydroxyproline residues [13]. In CLV3, the second hydroxyproline (Hyp) is arabinosylated and the three L-arabinose chain via linear β-1, 2 linkages enhances its receptor binding and function [14]. Some synthetic CLE peptides have been shown to influence and arrest root growth while others have no influence on root growth (9). Further studies with either synthetic peptide application, in vivo overexpression or disruptive CLE mutant plants have shown that some CLE peptides, including CLV3, function to define the delicate balance between stem cell proliferation and differentiation in SAM (shoot apical meristem) or RAM (root apical meristem) [9, 10, 15]. On the other hand, the other CLE peptides promote the development of procambial cells and suppress xylem differentiation in vascular tissues but do not arrest the development of SAM or RAM [12, 16]. Some CLE peptides can act synergistically with other CLE peptides to stimulate vascular cell proliferation [11]. In the group of CLE peptides with the first residue as a histidine, CLE41 and CLE44 both encode the same CLE peptide treachery element differentiation inhibitory factor (TDIF) [16].

In contrast to the dozens of plant peptide hormones identified so far, only a limited number of cognate cellular receptors have been identified that specifically perceive and becomes activated by these peptide ligands. Genetic analyses of *Arabidopsis* have shown putative sequences that can encode receptor like kinases (RLKs) which govern the overall signaling network in *Arabidopsis* by sensing extracellular cues and regulating gene expression [3]. Most of the known secreted peptide ligand receptors belong to the leucine-rich repeat receptor-like kinase (LRR-RLK) family of membrane integral receptors, which contains more than 200 members in *Arabidopsis* and makes up the largest family of plant receptor kinases[3, 7, 17, 18]. For example, the LRR-RLK protein CLAVATA1 (CLV1) has been identified as the plasma membrane receptor for CLV3 [15, 19–21]. LRRs form a β structure/ β turn that can create a surface for protein-protein interaction [22]. Previously it was assumed that plant LRRs form a structure similar to that of horseshoe solenoid [22], but recent studies have shown that plant LRR-RLKs with GxIP motif tend to adapt a super helical structure [23].

Two CLE genes, CLE41 and CLE44, encode a CLE peptide which is hydroxylated and proteolytically processed into a 12 amino acid residue peptide, known as tracheary element differentiation inhibitory factor (TDIF) [12]. The cognate receptor for TDIF peptide is TDR/PXY (TDIF RECEPTOR / PHLOEM INTERCALATED WITH XYLEM), which is also a LRR-RLK sharing 42% sequence identity with CLV1. Various scientific studies have confirmed the specific physical interaction between this particular ligand and its corresponding receptor [16, 24]. In the vascular tissues of *Arabidopsis*, phloem cells secrete TDIF which acts on procambial cells to promote the cellular proliferation and suppress the xylem differentiation via distinct downstream signaling pathways [16, 24]. A WUCHSCHEL-related HOMEOBOX 4 transcription factor (WOX4) is a downstream component of the TDIF/TDR signaling pathway that leads to cell proliferation but not to xylem differentiation in vascular tissues [25]. In addition, members of GSK3, BIN1, SKI, and SKII regulate the transcription factor BES1 to influence xylem differentiation but not during procambial cell proliferation [26]. How TDIF activates TDR to differentially regulate two separate processes remains elusive.

Most of the CLE peptides share high sequence identity (S1 Fig). How specific interactions can occur between similar kinds of CLE peptides and different LRR receptors is a long puzzling question. Recently two papers have reported the structural studies of TDIF-TDR(PXY) interactions. In addition, structure-guided mutational studies in both reports have indicated a conserved binding mode between CLE peptides and their cognate LRR-RLK receptors centered on residue G6 of CLEs [27, 28]. However, further studies are entailed in order to understand the binding specificities of CLE ligands to different LRR-RLKs. In this study, we use TDIF-TDR interaction as an example to understand the structural basis of differential CLE peptide recognition by LRR-RLKs in plant growth and development. Our results not only corroborate the previous structural studies of TDIF-TDR(PXY) interactions, but also suggests a differential binding mode of CLEs/LRR-RLKs interactions. In addition, the results will help to reveal how short peptide ligands specifically activate different LRR-RLKs in general.

## Results and discussion

### 1. Overall structure of the TDR-TDIF complex

**Fig 1:**
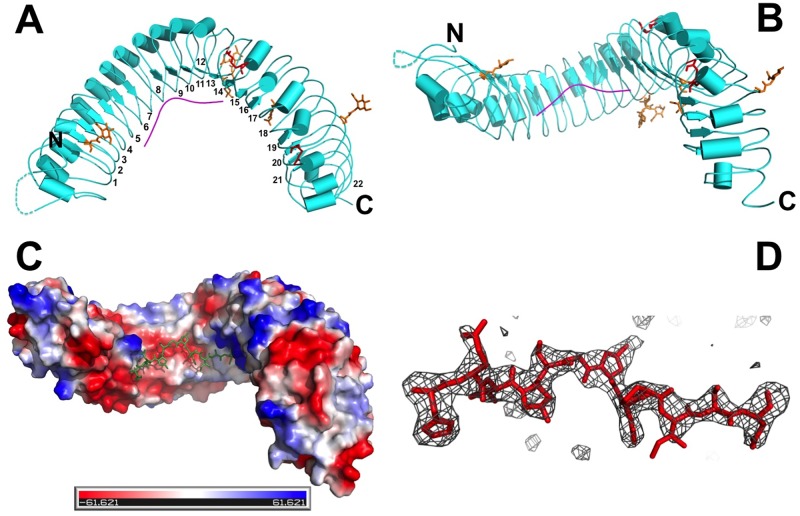
Structure of the extracellular domain of TDR and TDIF complex. (A) Top view of the TDR-TDIF structure in cartoon representation with smoothed loops. The disordered region in TDR structure (residues 48–56) is drawn in dotted line. Five glycosylated asparagine residues and the four cysteine residue that form two disulfide bonds between four neighboring leucine-rich repeats of TDR are depicted in stick representation and colored in orange and red, respectively. The structure of the TDR structure is shown in cyan, and the TDIF peptide structure is colored in red. The LRR repeat numbers are indicated on the structure. (Negatively charged surface is shown in red, and positively charged surface is depicted in blue). (B) Side view of the TDR-TDIF structure showing the superhelical S shaped structure. (C) Surface representation of the TDR-TDIF complex structure; positively charged surface is depicted in blue, and negatively charged surface is colored in red. (D) The TDIF peptide structure enveloped in a f_o_-f_c_ difference map contoured in 3 δ.

**Fig 2:**
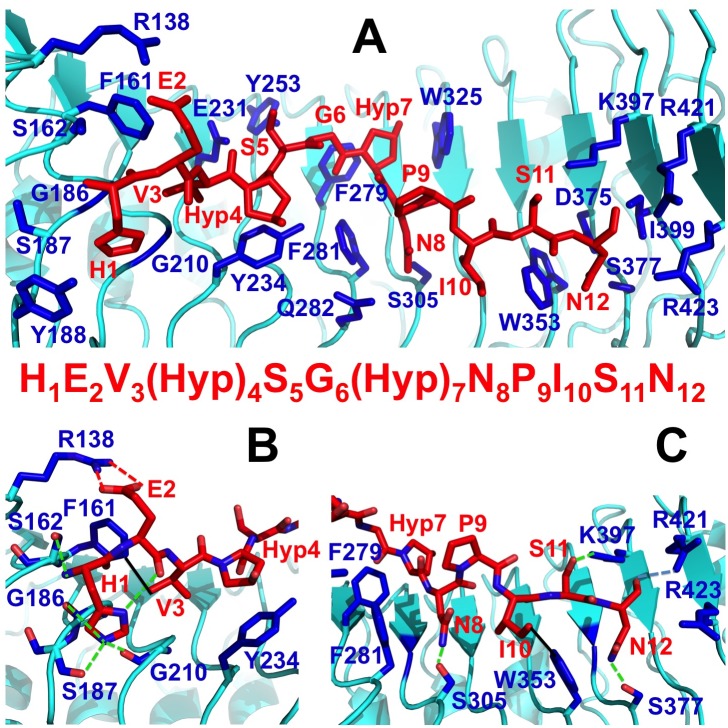
Binding interface between TDR and TDIF. (A) Surface residues of TDR that make contacts with TDIF peptide are depicted in blue, with their side chains shown in stick representation and residue numbered indicated. The rest of the TDR structure is colored in cyan, and the structure of TDIF peptide is shown in red and stick representation, with each residue number indicated. The sequence of the TDIF is shown at the bottom of the panel. (B) The N-anchoring site of TDIF on TDR. Hydrogen bonds are shown in green dotted lines, salt-bridge is depicted in a red dotted line, and hydrophobic interaction is shown in a solid black line. (C) C-anchoring site of TDIF on TDR. Hydrogen bonds are shown in green dotted lines and hydrophobic interaction is shown in a solid black line.

We have crystallized the extracellular domain of *A*. *thaliana* TDR (ecdTDR) alone and ecdTDR in complex with a synthetic TDIF peptide. Both crystals have the same space group p4_1_ and almost identical unit cell dimensions and crystal packing (Table 1). We have determined the apo-TDR structure by molecular replacement using the FLS2-ECD structure (PDB ID 4MN8) as an initial search model, and the structure of the ecdTDR/TDIF complex structure was then solved by molecular replacement using the apo-TDR structure as the search model. The atomic coordinates and structure factors of TDR and TDR-TIDF complex have been deposited in the Protein Data Bank under accession codes 5JFK and 5JFI respectively. The overall architecture of ecdTDR adopts an “S” shaped superhelical structure consisting of 22 LRRs (Fig 1), which resembles the other known plant LRR-RLK structures [29–32]. Almost all the 22 LRRs (except LRR18) in the TDR structures have a unified length of 24 amino acids with no variable insertion sequences. The conserved motif is “LXXLXLXXNXL/FXGXΦPXXΦXXLXX”, in which “X” stands for any residue and “Φ” stands for a hydrophobic residue (S3 Fig). Two pairs of cysteine residues, C390-C416 and C511-C535 form two disulfide bonds that tighten the parallel packing between LRR13-LRR14 and LRR18-LRR19 (S3 and S4 Figs). Five asparagine residues, N111, N356, N378, N471 and N525, are found to be N-glycosylated in the TDR structures. However, only one GlcNAc sugar residue on each site is visible in the electron density maps of the structures (S3 and S4 Figs). The TDIF peptide is bound on the concave surface of the TDR receptor, which stays on the middle of the surface and covers from LRR3 to LRR15. Each of the 12 residues of TDIF is visible in the electron density map, and the peptide adopts a fully extended conformation while making a blunt turn at the sixth residue G6 (Figs 1, 2 and S2 Fig). Superposition of our TDR-TDIF complex structure with the recently reported structures (PDB ID: 5GIJ and 5GR9) have resulted in a root mean square deviation (RMSD) of 0.596 and 0.682 over 594 residues respectively (S5 Fig), showing that these three ligand-receptor complex structures strongly agree with each other. However, in both our TDR apo-structure and the TDIF-TDR complex structure, the N-terminal (residues 40–81) and the C-terminal portions (residues 617–625) of the structures have poor electron density, indicating that those parts of the structures are flexible in the crystals. The rest of the structures are identical to the two previously published structures. The three TDIF ligand structures are superimposable, except that the side chain of E2 in our structure has about 150 degree rotation of the γ-carbon bond away from the structure solved by Zhang, et al., 2016 (PDB ID: 5GIJ), while it agrees with the structure of Morita, et al., 2016 (PDB ID: 5GR9) (S5 Fig).

**Table 1:** Crystallographic statistics.

	Apo TDR	TDR-TDIF complex
**Data collection**	Native	Native
Beam line	APS 22-ID	APS 22-ID
Wavelength (Å)	1.0000	1.0000
Space group	P4_1_	P4_1_
Cell dimensions a, b, c (Å) α, β, γ (°)	92.336, 92.336, 250.127, 90.00, 90.00, 90.00	92.445, 92.445, 252.615, 90.00, 90.00, 90.00
Resolution (Å)	50–2.65 (2.7–2.65)	50–2.75 (2.8–2.75)
Unique reflections	56517 (4100)	54275 (5006)
Redundancy	3.1 (1.8)	3.8 (2.2)
Data coverage (%)	93.1 (64.9)	98.9 (88.5)
I/σ	17.2 (2.0)	20.5 (1.6)
CC1/2	0.997 (0.683)	0.996 (0.702)
R_meas_ (%)	7.8 (61.1)	10.9 (59.3)
R_pim_ (%)	4.2 (43.7)	5.3 (35.3)
R_merg_ (%)	6.5 (46.2)	7.3 (41.5)
**Refinement**		
Resolution range (Å)	46.168–2.647	46.223–2.749
No. Reflections	53447 (3347)	52019 (4269)
No. Atoms	9211	9386
R_work_ (%)	22.17 (29.38)	21.45 (30.74)
R_free_ (%)	24.97 (35.25)	26.29 (39.60)
Mean B-factor (Å^2^), overall	63.3	65.1
R.m.s.d deviations		
Bonds (Å)	0.005	0.006
Angles (°)	1.102	1.211
Ramachandran Plot		
Favored (%)	87.00	86.11
Allowed (%)	11.17	11.84
Outliers (%)	1.83	2.06

Highest resolution shell is shown in parenthesis.

Each asymmetric unit contains two TDR molecules. There are two packing TDR dimers in the crystals (S6 Fig). The first packing interface buries 406.9 Å^2^ and the second one buries 580.9 Å^2^ surface area, corresponding to 1.7% and 2.4% of the total surface area of the TDR structure respectively, which is not sufficient to form a stable dimer in solution [33]. More importantly, each TDIF peptide only contacts an ecdTDR and does not bridge its dimerization. This clearly indicates that TDIF binding does not induce dimerization of TDR-ECD. The root mean square deviation between the apo-TDR and the TDR in the TDR-TDIF complex is 0.224 (S4 Fig), showing that ligand binding does not trigger significant conformational change in TDR. This confirms the previous structural observations [27].

### 2. Binding interface between TDIF and TDR

The TDIF peptide binds on the inner surface of TDR (Figs 1 and 2) that is identical to the previously reported structures [27, 28]. We have identified the following surface residues of TDR that make contacts with the TDIF peptide (Fig 2A): R138, F161, S162, G186, S187, Y188, G210, E231, Y234, Y253, F279, F281, Q282, S305, W325, W353, D375, S377, K397, I399, R421, and R423. Based on the structural observation, the peptide is bound by the following major interactions: (1) R138, F161, S162, G186, S187, and Y188 of TDR interact with H1, E2 and V3 of TDIF to anchor the N-terminus of the peptide; (2) E231, Y234, Y253, F279, F281 and Q282 of TDR bind the middle region of the peptide; (3) S305, W325, W353, S377, K397, I399, R421, and R423 of TDR bind to the N8, I10, S11 and N12 of the peptide to immobilize the C-terminus (Fig 2). Most of the above residues are highly conserved in TDR orthologs in other plant species, except for R138 that can be replaced with a histidine (S8 Fig). In four TDR paralogs of *A*. *thaliana*, CLV1, BAM1, BAM2 and BAM3, some of the residues that are critical for binding TDIF are also conserved. However, R138, F161, S162, S187, E231, Y253, F281 and K397 are variable, possibly due to the need to meet differential CLE peptide binding specificity of these LRR-RLKs (S9 Fig).

**Fig 3:**
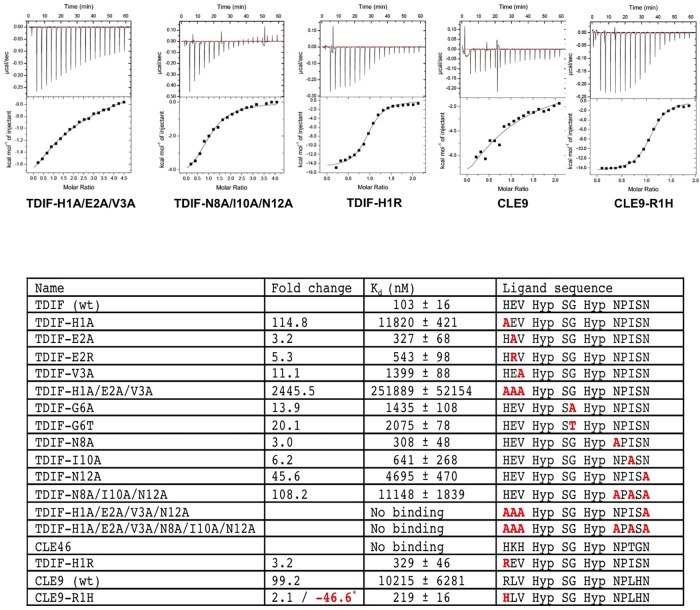
ITC measurements between wtTDR and TDIF mutants, CLE46, CLE9, and CLE9-R1H peptides. Representative ITC measurements between wild-type ecdTDR protein and TDIF-H1A/E2A/V3A, TDIF-N8A/I10A/N12A, TDIF-H1R, CLE9, and CLE9-R1H peptides are shown on the top panel; significant differences (greater than 2.5 fold) in the binding energy (dissociation constant Kd) between the wild type TDIF peptide and the mutants are summarized in the table below. In parallel, the calculated fold changes in K_d_ between wild type TDIF peptide and TDIF mutants along with CLE9 peptides are shown in a separate column. In comparison with wild type CLE9 and and CLE9-R1H mutant an additional fold change was found and is shown is red with a *. The sequence of each peptide is shown in the table, with the substituted resides colored in red.

The TDIF peptide docks on a shallow groove formed by the contacting residues of TDR (Figs 1C and 2A). The groove extends to the N-terminus of the peptide and is closed toward the C-terminus of TDIF. This structural arrangement is crucial for the peptide binding just fitting the groove of the interaction surface. Any extension in the C-terminus of the peptide might have an adverse effect on the peptide binding. In fact, TDIF-R or TDIF-H has been shown to have a weaker binding affinity than the wild type peptide [12]. Surface complementarity plays an important role in TDIF binding. Among the conserved interacting residues of TDR, F279 obstructs the peptide to make a turn after G6 of TDIF. G6 is conserved in most CLE peptides, and previous mutational studies with CLV3 have confirmed that G6 is critical for its interaction with TDR and *in vivo* function [27, 34, 35]. Our Isothermal Titration Calorimetric (ITC) measurement of a G6A TDIF mutant peptide has about 14 times weaker binding than the wild type peptide (Fig 3). The side chains of the three residues around G6, Hyp4, S5, and Hyp7 direct upwards and do not make significant contacts with TDR. Accordingly, alanine substitution mutations of these residues do not have a significant effect on TDR binding (Fig 3 and S10 Fig).

### 3. N- and C-terminal anchoring sites of TDIF

There are two major anchoring sites on the TDIF peptide that are separated by Hyp4-S5-G6-Hyp7 (Fig 2). The N-terminal anchoring site contains H1E2V3, and alanine substitutions at any of these residues significantly lower the binding affinity (Fig 3 and S10 Fig). H1A mutant has the strongest effect; with its binding affinity about 115 times lower than wild type peptide, which agrees with the reported in vivo studies [12]. The side chain of H1 dips into a binding pocket of TDR formed by residues S162, G186, S187, Y188 and G210. In addition, hydrogen bonding interactions create major interactive forces that significantly contribute to the binding energy of the N-terminal anchoring site between the δ1-NH group of H1 and carbonyl groups of G186, S187, and G210, ε1-NH group of H1 and carbonyl group of E2, and carbonyl group of H1 and hydroxyl group of S162 (Fig 2B). The E2A mutant has a weaker effect (3.2-fold increase in K_d_) on TDIF binding; while a E2R substitution by reversing the charge on E weakens the binding further to 5.3-fold, indicating that ionic interaction between E2 of TDIF and the TDR receptor plays a role in its binding. On the other hand, the interacting residue R138 of TDR can be substituted by a histidine in its orthologs (S8 Fig), indicating that the electrostatic interaction is not crucial for TDIF binding. However the reported in vivo study of E2A mutant has no significant effect on the inhibition of tracheary element differentiation, which might be caused by the differences in either in vitro or in vivo assays [12]. V3 of TDIF forms a hydrophobic interaction with the side chain of F161. Consequently, a V3A mutant TDIF peptide has a binding affinity with TDR 11.1 times lower than the wild type. Triple alanine-substitution mutant of TDIF-H1A/E2A/V3A has a negligible binding affinity to TDR when compared to the wild type, demonstrating that the N-terminal anchoring site plays a dominant role in TDIF binding to TDR (Figs 2B and 3). Altogether, the N-terminal anchoring site H1E2V3 immobilizes the peptide on the TDR receptor.

Three TDIF residues N8, I10 and N12 form a C-terminal anchoring site (Fig 2C). Alanine substitutions of these residues N8A, I10A and N12A radically weaken their binding with TDR receptor. δ-NH2 group of N8 forms hydrogen bonds with the hydroxyl group of S305; side chain of I10 forms hydrophobic interaction with W353; δ-NH2 group of N12 forms hydrogen bonds with the hydroxyl group of S377 and carbonyl group of N12 forms a hydrogen bond with the side chain of R421. When all three residues were replaced with alanine, the mutant peptide (N8A/I10A/N12A) had a very weak affinity of about 11 μM, almost 110 times lower than the wild-type peptide, indicating that along with the N terminal anchoring site, the C-terminal anchoring site of the peptide also plays a critical role in its binding to TDR. Mutating both the N-anchoring site and the C-terminal anchoring site residues H1A/E2A/V3A/N8A/I10A/N12A, or H1A/E2A/V3A/N12A, resulted in no binding of the peptides to TDR. In the structure, hydrogen-bonding interaction between side chains of S11 and K397 has been observed (Fig 2C). However, S11A substitution shows that S11 does not play a significant role in TDIF-TDR binding as it barely affects the binding affinity in comparison to the wild type TDIF. The side chain of P9 points upward without making any contact with TDR and an alanine substitution of P9A has only a minor effect (2 fold decrease) on TDR binding, which is likely caused by the conformational change in the mutant peptide. However, a reported in vivo study of the TDIF-P9A mutant has a significant in effect on tracheary element differentiation inhibition [12]. Different assay system could have resulted in this observed discrepancy. In agreement with a previous report, CLE42 peptide, in which the E2 has been replaced with glycine, showed stronger binding to TDR than wild type TDIF [12]. However, for CLE46, after changing residues in both the N- and C-terminal anchoring sites, no measurable binding was detected by ITC (Fig 3 and S10 Fig). In general, our results agree with the previously reported mutational studies of these residues [12, 27].

The linker region between the N- and C-terminal anchoring sites of TDR contains Hyp4-S5-G6-Hyp7, which is highly conserved in most CLE peptides (S1 Fig). We did not observe significant contacts between side chains of Hyp4, S5, Hyp7 and TDR (Fig 2). In fact, alanine substitution mutant of each and proline substitution of either Hyp residue demonstrated only minor changes in their binding affinities. The linker region residues of CLE3 have been previously studied by mutational analyses, which showed that only G6 plays an essential role in CLE/LRR-RLK binding and in vivo function [34]. Indeed, both G6A and G6T mutant peptides have 13.9 and 20.1-fold lower affinity than wild type respectively, indicating that only G6 plays an essential role in CLE/LRR-RLK binding in vitro (Fig 3 and S10 Fig). In the structure of the TDR-TDIF complex, TDIF-G6 makes contact with TDR-F279, which pushes the peptide to make a blunt turn. The flexibility conferred by G6 to the peptide may be required for TDIF to adopt the observed binding configuration to accommodate both the N- and C- terminal anchoring sites binding. Again, our results are in concord with the previous studies [12, 27].

### 4. TDIF binding sites on TDR

**Fig 4:**
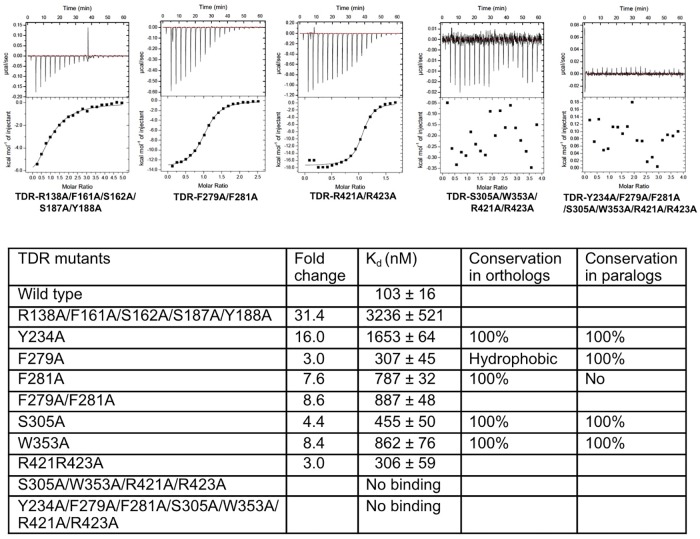
ITC measurements between wild type TDIF and ecdTDR mutants. Representative ITC measurements between wild type TDIF peptide and five ecdTDR mutants are shown on the top panel; dissociation energy (K_d_) values with significant difference (greater than 2.5 fold) in binding energy between the wild type and mutant ecdTDR proteins are summarized in the table below. In parallel, the calculated fold changes in K_d_ between wild type ecdTDR and ecdTDR mutants are shown in a separate column. Sequence conservation of each mutated residue in 8 TDR orghologs or in 5 TDR paralogs are listed along with their conservation percentages For both orthologs and paralogs, some of the variant residues are indicated.

To assess the energy contributions of the side chains of each interacting residue on TDR to TDIF binding, we made alanine substitution mutants of TDR and measured TDR mutant/TDIF binding affinity by isothermal titration calorimetric (ITC) (Fig 4 and S11 Fig). In addition, we observed a specific electrostatic interaction between R138 of TDR and E2 of the TDIF peptide. Thus, to test whether abolishing the interaction would influence binding energy, we made an R138E mutant and found that by reversing the charge does not result into significant difference in binding energy. (Fig 4 and S11 Fig). We observed that R138, F161, S162, G186, S187, and Y188 of TDR interact with TDIF N-terminal anchoring site (Fig 2B). However, a single-site mutation of each of these residues (R138A, S162A, G186A, S187A, Y188A) has either no adverse effect or mild effect (F161A) on TDIF binding. The F161A and S162A mutants have relatively stronger effects on TDR binding in a previous GST pull-down assay [28], which again indicates that different assay systems could cause some discrepancy. When all five residues are replaced by alanine, the mutant TDR has a cumulative effect, with 31.4-fold decrease in its binding affinity to TDIF. R138 can be replaced by a histidine in TDR orthologs, but the other four residues are highly conserved (S8 Fig). TDR-R138 forms an electrostatic interaction with TDIF-E2, and mutating E2 has only a mild effect on TDIF-TDR binding. This shows that TDR-R138 is not critical for TDIF recognition. In contrast to the high conservation of these five residues in TDR orthologs, most of them are variable in TDR paralogs, such as CLV1 and BAM1, 2 and 3 (S9 Fig). This suggests that the CLE peptides N-terminal anchoring site-binding surface on plant LRR-RLKs are diversified to meet differential CLE binding specificity of each receptor.

In the structure of TDR-TDIF complex, S305, W325, W353, S377, K397, I399, R421, and R423 of TDR interact with the N8, I10, S11 and N12 of TDIF peptide to immobilize its C-terminal anchoring site (Fig 2C). We observed that TDR-S305 forms a hydrogen bond with the side chain of TDIF-N8. Accordingly, an alanine substitution mutation of TDR-S305 has 4.4-fold lower binding than the wild type TDR receptor. In addition, TDR-W353 interacts with the side chain of TDIF-I10 with a hydrophobic interaction and subsequently, a W353A substitution resulted in 8.4 fold lower binding to TDIF. Although the side chains of other residues in the binding surface of TDIF C-terminal anchoring site have made noticeable contact with TDIF, single alanine substitution mutation of each side chain residue did not significantly attenuate its binding to TDIF. TDR-R421 and R423 are the end cap residues that restrict the C-terminal end of TDIF, and the side chain of R421 forms a hydrogen bond with the side chain hydroxyl group of TDIF-N12. In order to assess the contributions of both the residues in peptide binding, we generated TDR-R421A/R423A double mutant and measured its binding with TDIF. The binding affinity is 3-fold lower than that of wild type TDR, higher than either of the single mutant. Intriguingly, TDR-S305A/W353A/R421A/R423A quadruple mutant has no measurable binding to TDIF, showing that the binding pocket in the C-terminal anchoring site plays a central role in TDIF binding by TDR (Fig 4 and S11 Fig). Although the R423A mutant showed an apparent weaker binding on TDR in a GST pull-down assay than our ITC measurement [28], in general, our results agree with the previous reports [27, 28].

In the TDIF binding groove on TDR, side chains of a series of aromatic residues, such as Y234, Y253, F279, F281, W325, and W353 of TDR, surround the main chain of the peptide along its winding path on the inner surface of the receptor (Fig 2A). In addition, TDR-E231 and Q282 also make contacts with the peptide. However, in comparison with wild type TDR, both E231A and Q282A mutants show similar binding strength to TDIF, indicating that they play minor roles in TDR-TDIF interaction. E231 is invariant in TDR orthologs, while Q281 is not conserved, and both residues are variable in TDR paralogs. Among the aromatic residues, Y253 is only conserved in TDR orthologs but not in its paralogs, and W325 can be replaced by a hydrophobic residue in their homologs (S8 and S9 Figs). However, mutational studies have shown that both residues do not contribute significantly to TDIF binding (Fig 4 and S11 Fig). The other three aromatic residues, Y234, F279, and F281, contribute significantly to the peptide binding as corroborated by mutational analyses. Furthermore, both F279 and F281 form a gate to bend the flexible hinge TDIF-G6 of the peptide. As such, simultaneously mutating both residues to alanine causes an 8.6-fold decrease in its binding to TDIF. Moreover, a combined adverse effect was observed when replacing Y234, F279, and F281 together with the C-terminal anchoring site binding residues, R421 and R423, to alanine. The resulting mutant receptor has no measurable binding to TDIF. Interestingly, most of the residues involved in binding of TDR for the TDIF C-terminal anchoring site and linker region are conserved in both TDR orthologs and paralogs, in contrast to the lower conservation of N-terminal anchoring site binding residues in TDR paralogs (S8 and S9 Figs). Combining our examination with previously reported mutational studies [27, 28], we hypothesize that the N-anchoring site binding pocket confers CLE recognition specificity to LRR-RLK receptors, while all interacting residues of TDR provide binding energy to peptide ligand interaction.

### 5. Implication for differential CLE peptide perception by plant receptors

CLE peptides share general sequence features, with the four residues (Hyp4-S5-G6-Hyp7) in the linker region highly conserved in most CLEs (S1 Fig). G6 has been previously shown to be essential for CLE3 functions in vivo [34]. In our mutational analysis, G6 of TDIF was shown to be required for binding to TDR (Fig 3). Interestingly, we observed in the complex structure that the side chain of TDR-F279 obstructs G6 in the binding groove, which requires a unique glycine residue to confer main-chain flexibility to fit this type of interaction (Fig 2A). In other TDR orthologs a hydrophobic residue such as leucine can replace F279 (S8 Fig), suggesting that it may function similarly in TDIF binding. However, it is invariant in TDR paralogs, such as BAM1, 2 and 3 and CLV1 (S9 Fig). Such similarities suggest a possibility that all CLE peptides may bind their respective plant LRR-RKs in a mode similar to TDR-TDIF interaction as also indicated in the previous studies, and that the G6 residue of CLEs dictates this binding feature [12, 27, 28, 34, 36]. In accordance with the previously published results, our mutagenesis studies demonstrate the linker region of CLEs is not essential for TDR binding. However, the roles of the two highly conserved Hyp residues remain unknown.

Almost all the residues on TDR that are essential to bind TDIF C-terminal anchoring site and the linker region are conserved in both TDR orthologs and paralogs (S8 and S9 Figs). However, the residues constituting the N-terminal anchoring site are only conserved in its TDR orthologs and are variable in paralogs except TDR-Y188 (S10 and S11 Figs). It is possible that the N-terminal anchoring site confers the binding specificities between CLEs and their cognate LRR-RLKs. Our ITC data of the TDIF mutants suggests, TDIF-H1 contributes most to the binding of the N-terminal anchoring site, while TDIF-E2 plays a marginal role in this interaction. H1 is conserved in TDIF, CLE42, and CLE46 (S1 Fig), which have been classified into the same group [11, 37, 38]. Changing E2 to G in CLE42 does not influence the binding significantly, while E2K, V3H, I10T and S11G substitutions in CLE46 render it unable to bind TDR (Fig 3). In all other CLEs, however, an arginine replaces histidine at the first position. It is conceivable that the first histidine residue dictates the receptor binding specificity of the H1 containing CLEs. In order to test this hypothesis, we created two CLE mutant peptides TDIF-H1R and CLE9-R1H and examined their binding to recombinant ecdTDR protein by ITC (Fig 3). R1H substitution of TDIF lowered its binding affinity to TDR by 3.2-fold, indicating that this mutation disturbs TDIF binding to TDR. Wild type CLE9 peptide has a very low binding affinity to TDR, while replacing R1 with a histidine residue significantly improves its binding to TDR, indicating that swapping arginine to histidine confers a non-TDIF CLE peptide into a TDIF binding specificity. These biochemical binding data support our hypothesis that the first residue of CLE peptides is critical for their binding specificity to their cognate receptors.

In conclusion, our structural analyses provide insights about the ligand perception and specific interactions between the CLE peptides and their cognate receptors. In addition, our in vitro biochemical studies have shown that the interactions between the ligands and the receptors at the C-terminal anchoring site provide conserved binding, while the binding interactions occurring at the N-terminal anchoring site dictate differential binding specificities between different CLE ligands and LRR-RLK receptors. Our studies will open new avenues to further understand how similar CLE ligands bind to various similar leucine-rich repeat receptor-like kinases (LRR-RLKs) to elicit differential signaling outcomes.

## Materials and methods

### Protein expression and purification

To elucidate the molecular basis of TDR-TDIF interaction, we expressed the extracellular domain of TDR (residues 30–642) from *Arabidopsis thaliana* using Bac-to-Bac baculovirus-mediated insect cell expression system (Invitrogen). DNA fragment encoding the *A*. *thaliana* TDR gene (residues 30–642) were amplified by polymerase chain reaction (PCR) from an *A*. *thaliana* Expressed Sequence Tag (EST) clone as template. The amplified DNA fragment was then cloned into a modified pFastBac1 vector with the secretion signal sequence of baculovirus gp67 glycoprotein fused to its 5’ end and an engineered 6-histidine tag at the carboxyl terminus of the TDR protein. The construct plasmid was used to transform DH10Bac (Invitrogen) competent cells to generated recombinant bacmid DNA. Then the bacmid DNA was used to transfect sf9 insect cells to generate recombinant virus which was then amplified three times to passage 3. We used 20 ml passage 3 virus to infect each liter of High Five cells with a cell density of 1.5 x 10^6^ in SF900 II SFM medium (Invitrogen). The recombinant protein was expressed in High Five cells at 27°C. After 60 hours the medium of the infected cells was collected and the secreted recombinant protein was first purified by Ni-NTA (Qiagen) affinity chromatography, and then further purified by size-exclusion chromatography in buffer containing 20mM Tris-HCl, pH 8.0, and 100mM NaCl. The purified protein was concentrated to 5mg/ml for crystallization. The TDR mutants were generated using a PCR-based site-directed mutagenesis kit (Invitrogen), and the mutant TDR proteins were expressed and purified as the same procedure of the wild-type TDR (30–642) protein.

### Crystallization and data collection

The bioactive TDIF peptide was chemically synthesized (peptide 2.0) with amino acid sequence HEV(Hyp)SG(Hyp)NPISN. The recombinant TDR protein was concentrated to 5mg/ml (42μM), and 420μM TDIF peptide was mixed and incubated at 4°C for 1 hour. Both the ecdTDR and ecdTDR/TDIF complex proteins were subjected to extensive crystallization screening. Both proteins were crystallized in P4_1_ crystal forms using both hanging drop vapor diffusion and sitting drop method at 18°C by mixing equal volumes of the purified protein and the crystallization condition of 200mM MgSO_4_ and 20% PEG 3350. For data collection, all crystals were flash frozen in the respective crystallization conditions supplemented with 25% (v/v) glycerol. Diffraction data were collected at the 22-ID (SERCAT) beam line of the Advanced Photon Source (APS). All diffraction data were processed using the HKL2000 suite [39] and their statistics are shown in Table 1.

### Structure determination, refinement and analysis

We have determined the apo-TDR structure by molecular replacement using the FLS2-ECD domain structure as an initial search model (PDB ID 4MN8). The model of ecdTDR structure was built in COOT [40], and refined with REFMAC5 [41] and PHENIX [42]. The structure of the ecdTDR/TDIF complex structure was then solved by molecular replacement using the refined model of the solved apo-TDR structure as the search model. Both crystals contain two TDR or TDR/TDIF complex molecules in each asymmetric unit cell. Both TDR structure models contain residues 40–625. Five asparagine residues (N111, N356, N378, N471 and N525) in both TDR structures are N-glycosylated and one GlcNAc sugar residue on each asparagine is visible. In addition to the observed N-glycosylation, four cysteine residues are observed to form two disulfide bonds between C390-C416 and C511-C535. The structures were analyzed using the CCP4 suite [43] and the PISA server [44], and the figures were made using PyMOL [45].

### Isothermal Titration Calorimetry (ITC) measurements

ITC experiments were performed on a MicroCal Auto-iTC200 instrument. 0.5–1 mM of each synthetic TDIF, CLE9, TDIF-H1R or CLE9-R1H peptide was titrated into a 10–40 μM solution of ecdTDR protein. Experiments were carried out at 25°C in a buffer containing 10 mM sodium citrate and 100 mM NaCl at pH 5.5. The data was analyzed using ORIGIN software. The association constant (K_a_), enthalpy change (**Δ**H), and the stoichiometry (N) were calculated by fitting the thermograms to one set of binding sites. The association constant (K_a_) was calculated by fitting the thermograms to one binding site. The dissociation constant (K_d_) was calculated using the equation K_d_ = 1/K_a_.

## Supporting information

S1 Fig Sequence alignment of the CLE peptides in *Arabidopsis thaliana*. The conserved proline residues which are potentially hydroxylated are shown in red, and the rest of the 12 conserved residues in the CLE motifs are shown in blue. The surrounding residues of CLE motifs are shown in black. The CLE motifs with the first residue as a Histidine are listed at the bottom. The length of each CLE protein is listed at the end of each line.
(TIF)

S2 Fig TDR-TDIF electron density maps. (A) 2f_o_-f_c_ map around the TDR-TDIF binding interface of the TDR-TDIF complex structure contoured in 1.5 δ. (B) A composite omit map of the TDIF peptide contoured in 2 δ.
(TIF)

S3 Fig Sequence alignment of the 22 Leucine-Rich Repeats (LRRs) in the extracellular domain of TDR. The conserved LRR motif of TDR is shown on the top of the alignment, with “X” stand for any residue, and “Φ” stands for a hydrophobic residue. The common secondary structure elements of the LRRs in TDR are placed on the top of the panel, with a blue arrow for b-strand, red cylinder for helix, and green line for loop. The turn in the LRR is indicated above the corresponding region of the loop. The conserved residues among LRR repeats of TDR are colored in red. The five glycosylated asparagine residues identified in the structures are colored in blue, and the four cysteine residues that form two disulfide bonds in the structures are colored in yellow and connected with yellow lines to indicate the formation of the disulfide bonds.
(TIF)

S4 Fig The five N-glycosylation sites on the extracellular domain of TDR. Only one Glc-NAc sugar residue on each of the five glycosylated asparagine residues is observed in the electron density maps of the structures. The two disulfide bonds between LRR13/LRR14 and LRR18/LRR19 are shown in stick representation with the residue numbers of the four cysteines indicated.
(TIF)

S5 Fig Superposition of TDR-TDIF complex structures. (A) Superposition of our TDR-TDIF complex structure (PDB ID: 5JFI, colored in cyan) with the recently reported structures (PDB ID: 5GIJ, colored in yellow, and 5GR9, colored in blue respectively). (B) Superposition of the TDIF peptide structures with the same color codes applied.
(TIF)

S6 Fig Two crystal packing dimers of the TDR-TDIF complex. The measured distances between the C-terminus of the two TDR monomers in each dimer and the packing interfaces are indicated.
(TIF)

S7 Fig Superposition of the TDR-TDIF (cyan) and the apo-TDR (red) structures. The root-mean-square deviation (rmsd) of the alignment is 0.224.
(TIF)

S8 Fig Protein sequence alignment of eight TDR orthologs. Ath stands for *Arabidopsis thaliana*; Csa, *Camelina sativa*; Bra, *Brassica rapa*; Tha, *Tarenaya hassleriana*; Mdo, *Malus domestica*; Atr, *Amborella trichopoda*; Sit, *Setaria italic*; Jcu, *Jatropha curcas*; respectively. Overall sequence identity between *Arabidopsis thaliana* TDR and the TDR of *Camelina sativa*, *Brassica rapa*, *Tarenaya hassleriana*, *Malus domestica*, *Amborella trichopoda*, *Setaria italic*, *Jatropha curcas* is 89%, 84%, 78%, 60%, 56%, 50%, 46%, respectively. Residue numbers of *A*. *thaliana* TDR are indicated on the top the sequences. Each LRR repeat is indicated on the top of the sequences. The conserved TDIF interacting residues of TDR are shown in red, and the cysteine resides which form disulfide bonds in the TDR structures are depicted in dark yellow, and the five observed N-glycosylation site asparagine residues are colored in blue. The consensus residues are shown below each alignment.
(TIF)

S9 Fig Protein sequence alignment of *Arabidopsis thaliana* TDR with four paralogs, CLV1, BAM1, BAM2 and BAM3. Residue numbers of *A*. *thaliana* TDR are indicated on the top the sequences. Each LRR repeat is indicated on the top of the sequences. The conserved TDIF interacting residues of TDR are shown in red, and the conserved cysteine resides which form disulfide bonds in the TDR structure are depicted in dark yellow, and the conserved N-glycosylation site asparagine residues are colored in blue. The consensus residues are shown below each alignment.
(TIF)

S10 Fig ITC measurements of wtTDR withTDIF mutants, CLE42, and CLE46. K_d_ values of the wtTDR with the TDIF mutants, CLE42 and CLE46 are shown along with their sequences in the table below.
(TIF)

S11 Fig ITC measurements between wild type TDIF and ecdTDR mutants. The measurements of the binding interactions (K_d_ values) between wtTDIF and ecdTDR mutants are shown in the table below. The sequence conservation of ecdTDR between orthologs and paralogs are also shown in the table. There are some residues that are not entirely conserved such as R138 which can be either R and H in orthologs and either R, N, S in paralogs.
(TIF)
