# Supplementary figures and images for: Differential CLE peptide perception by plant receptors implicated from structural and functional analyses of TDIF-TDR interactions
Source: PLoS One. 2017 Apr 6;12(4):e0175317. doi: 10.1371/journal.pone.0175317 (PMC5383425; doi:10.1371/journal.pone.0175317)

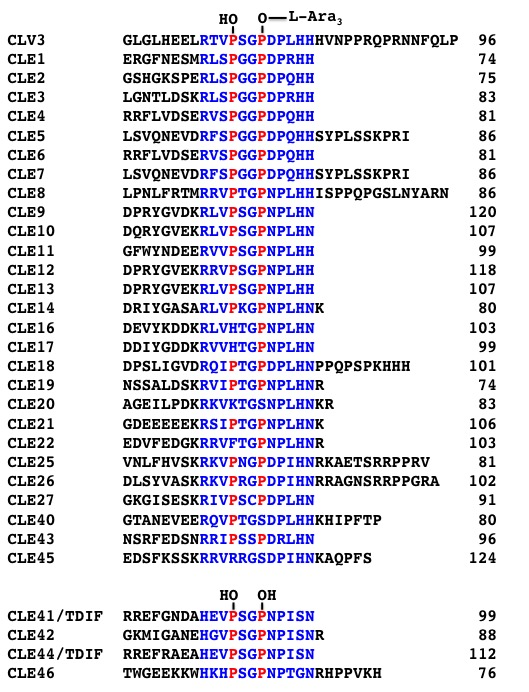

Supplement: S1 Fig — The conserved proline residues which are potentially hydroxylated are shown in red, and the rest of the 12 conserved residues in the CLE motifs are shown in blue. The surrounding residues of CLE motifs are shown in black. The CLE motifs with the first residue as a Histidine are listed at the bottom. The length of each CLE protein is listed at the end of each line. (TIF) [file pone.0175317.s001.tif]

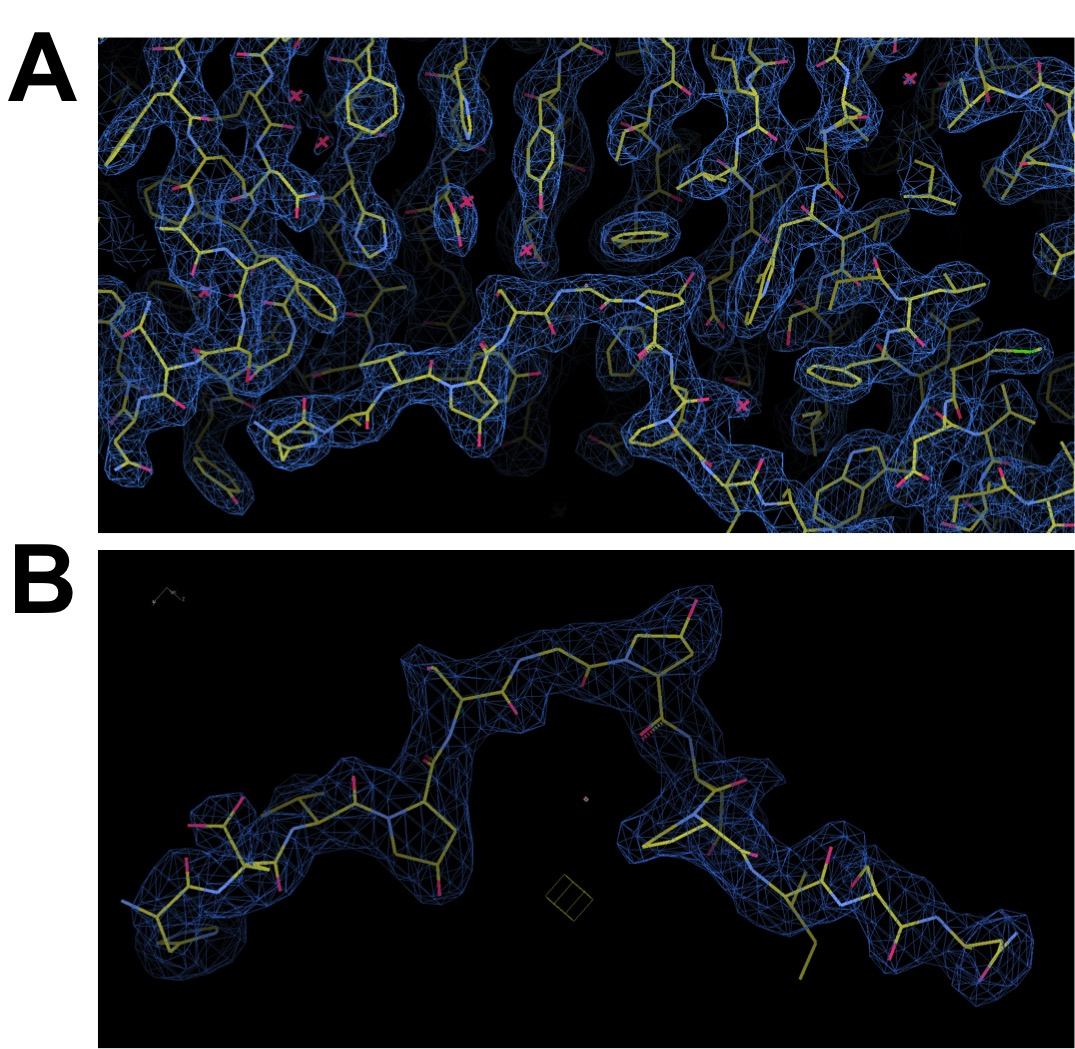

Supplement: S2 Fig — (A) 2fo-fc map around the TDR-TDIF binding interface of the TDR-TDIF complex structure contoured in 1.5 δ. (B) A composite omit map of the TDIF peptide contoured in 2 δ. (TIF) [file pone.0175317.s002.tif]

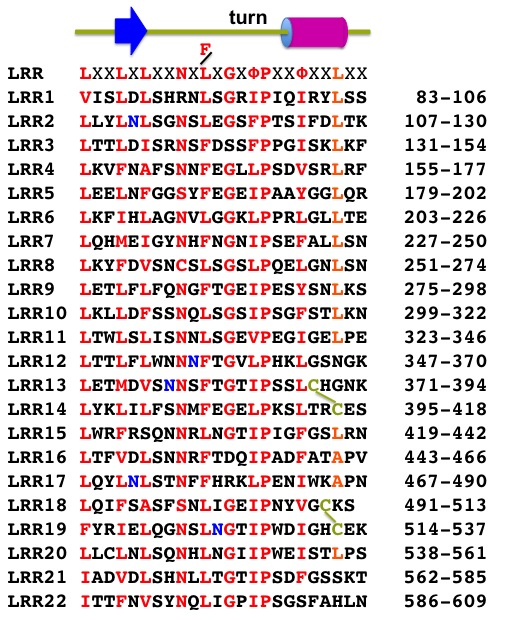

Supplement: S3 Fig — The conserved LRR motif of TDR is shown on the top of the alignment, with “X” stand for any residue, and “Φ” stands for a hydrophobic residue. The common secondary structure elements of the LRRs in TDR are placed on the top of the panel, with a blue arrow for b-strand, red cylinder for helix, and green line for loop. The turn in the LRR is indicated above the corresponding region of the loop. The conserved residues among LRR repeats of TDR are colored in red. The five glycosylated asparagine residues identified in the structures are colored in blue, and the four cysteine residues that form two disulfide bonds in the structures are colored in yellow and connected with yellow lines to indicate the formation of the disulfide bonds. (TIF) [file pone.0175317.s003.tif]

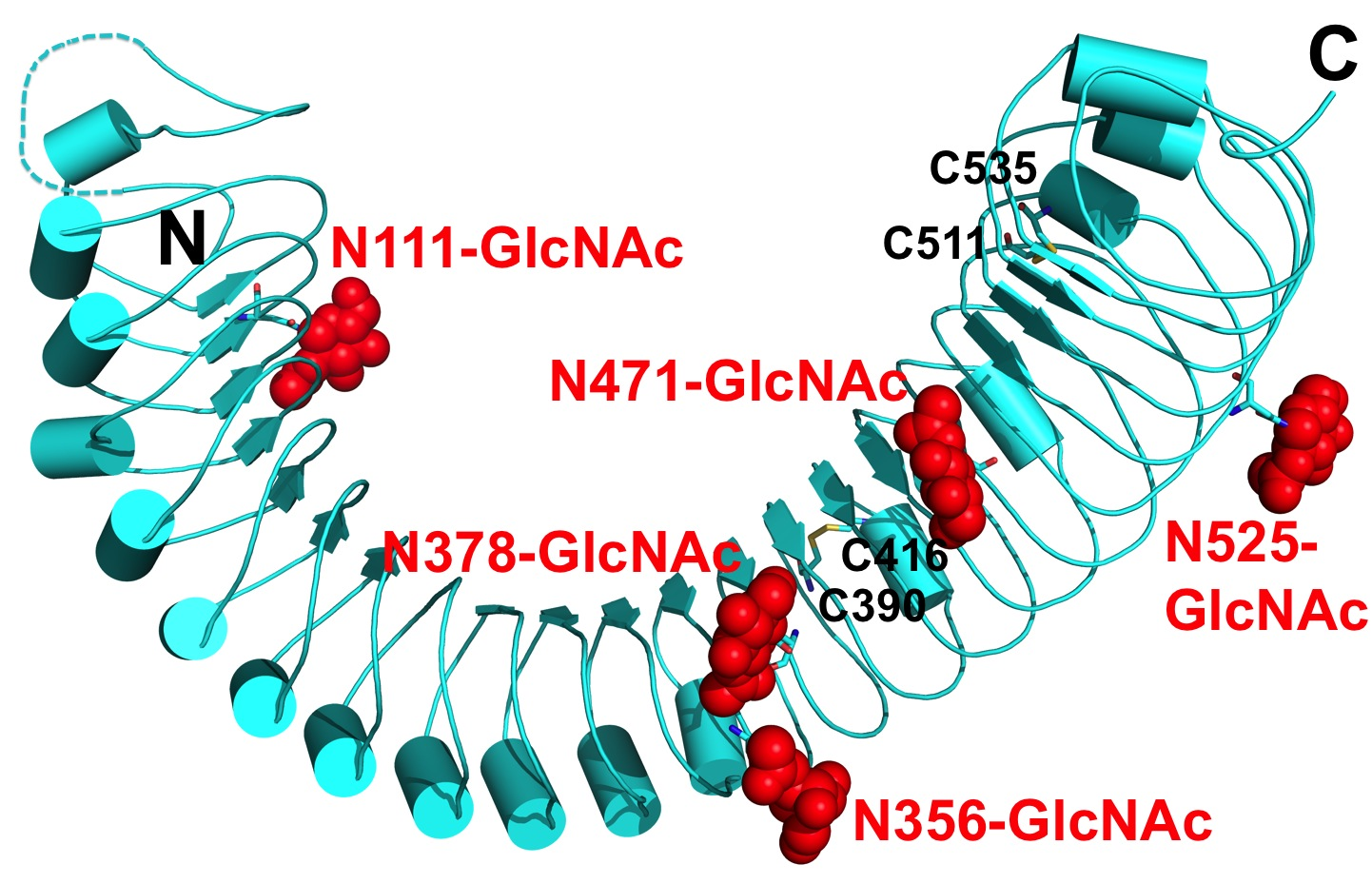

Supplement: S4 Fig — Only one Glc-NAc sugar residue on each of the five glycosylated asparagine residues is observed in the electron density maps of the structures. The two disulfide bonds between LRR13/LRR14 and LRR18/LRR19 are shown in stick representation with the residue numbers of the four cysteines indicated. (TIF) [file pone.0175317.s004.tif]

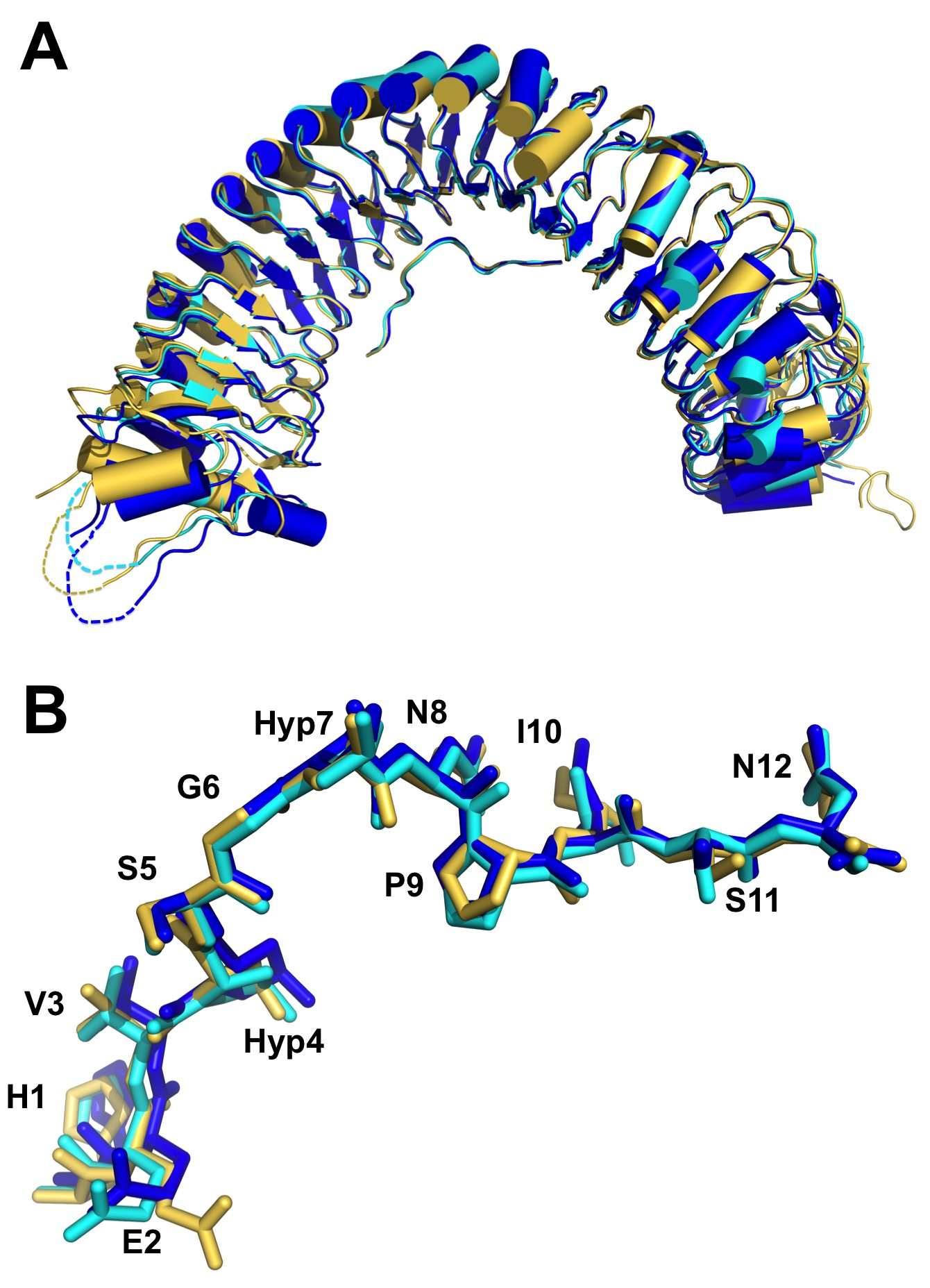

Supplement: S5 Fig — (A) Superposition of our TDR-TDIF complex structure (PDB ID: 5JFI, colored in cyan) with the recently reported structures (PDB ID: 5GIJ, colored in yellow, and 5GR9, colored in blue respectively). (B) Superposition of the TDIF peptide structures with the same color codes applied. (TIF) [file pone.0175317.s005.tif]

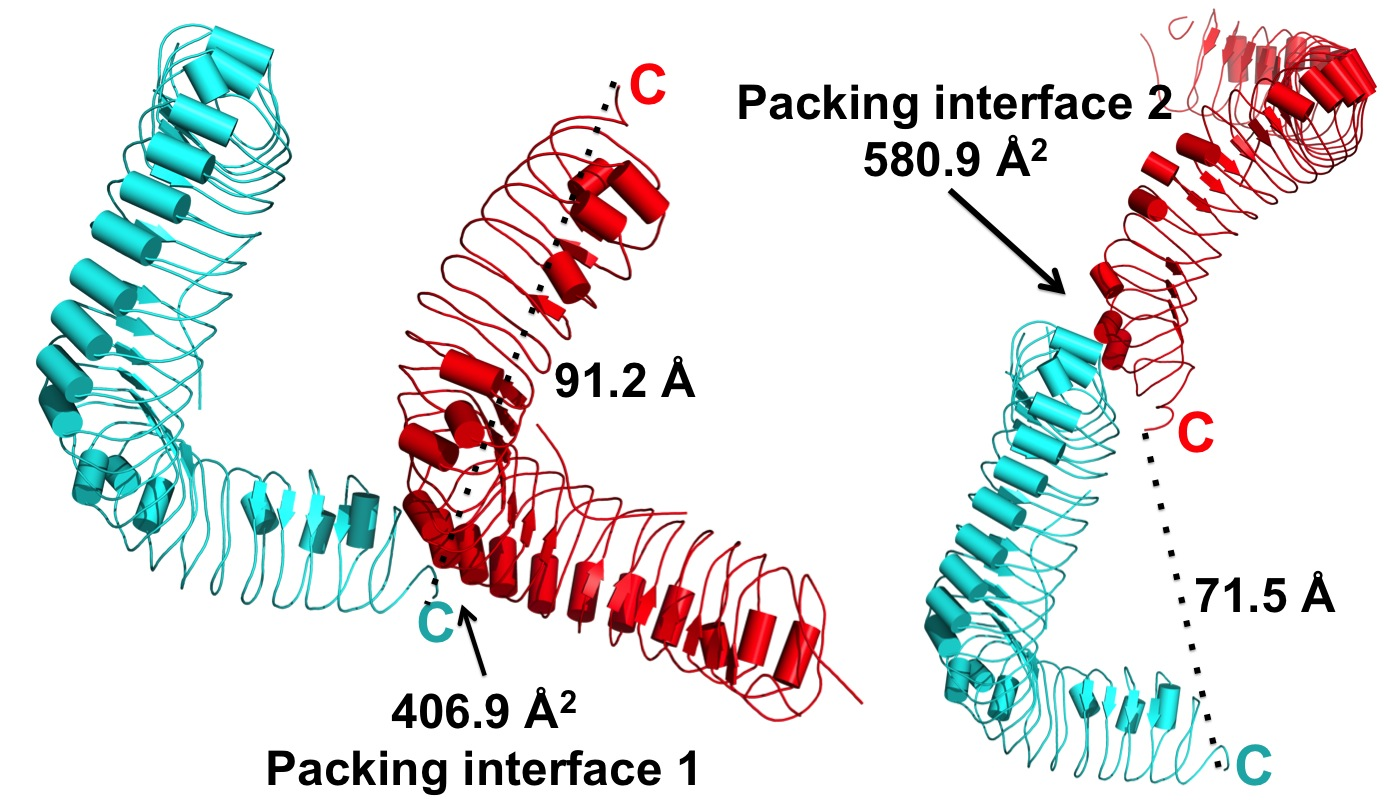

Supplement: S6 Fig — The measured distances between the C-terminus of the two TDR monomers in each dimer and the packing interfaces are indicated. (TIF) [file pone.0175317.s006.tif]

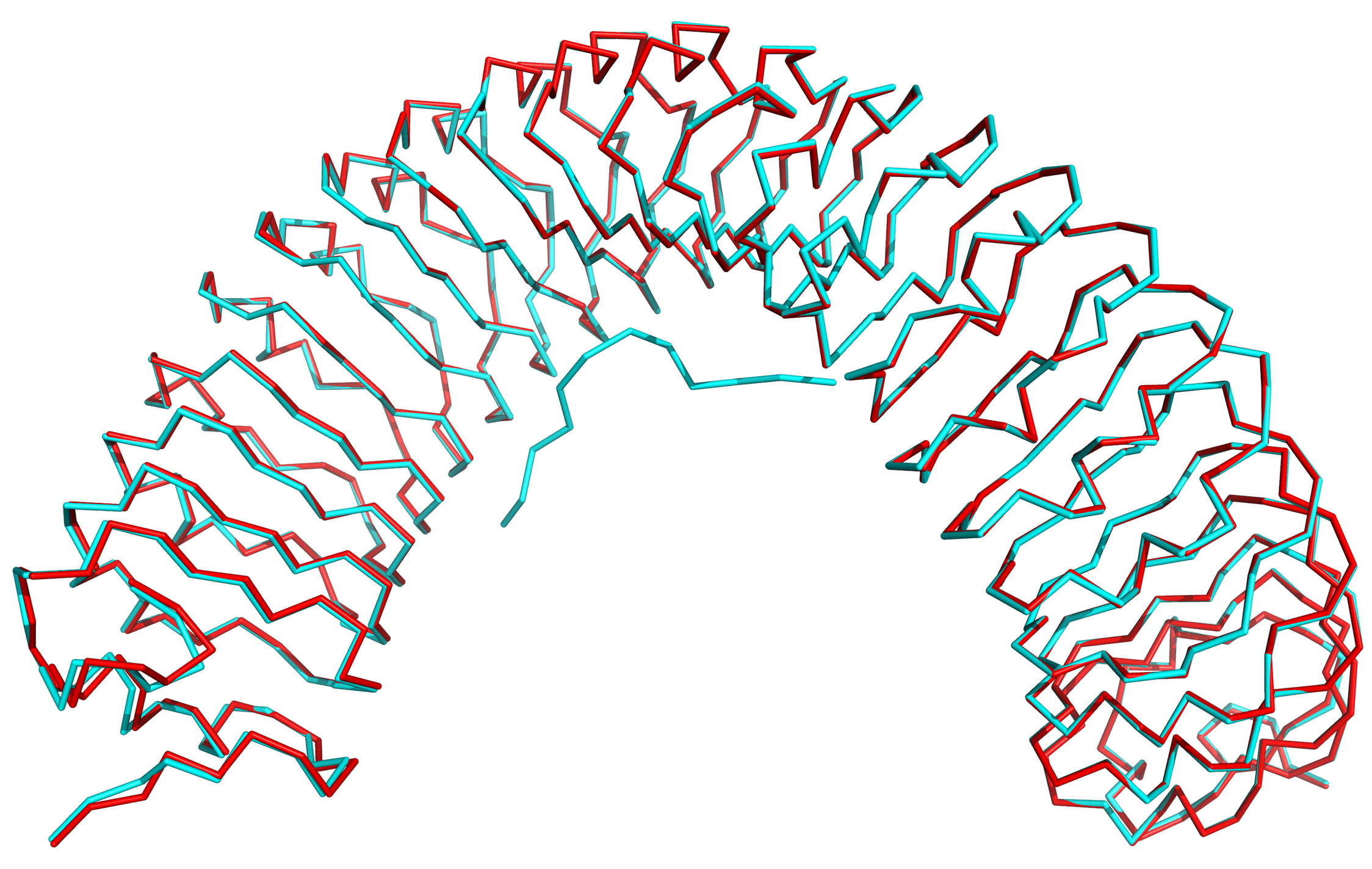

Supplement: S7 Fig — The root-mean-square deviation (rmsd) of the alignment is 0.224. (TIF) [file pone.0175317.s007.tif]

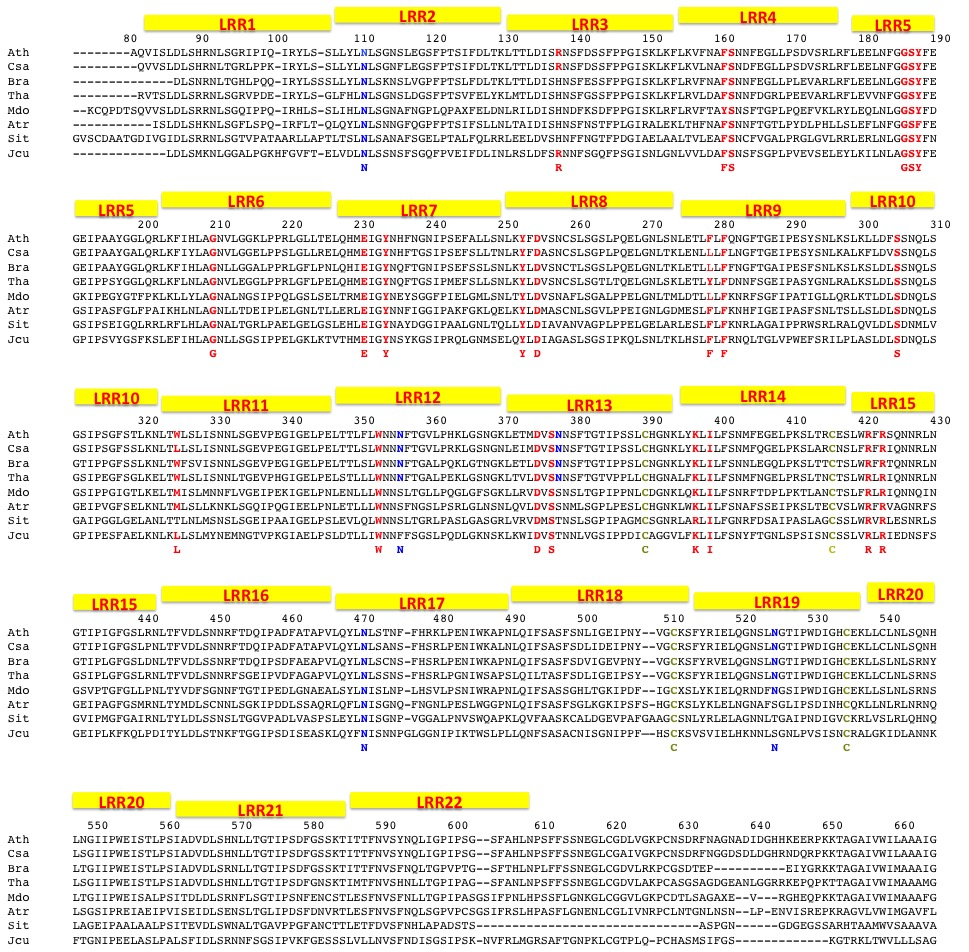

Supplement: S8 Fig — Ath stands for Arabidopsis thaliana; Csa, Camelina sativa; Bra, Brassica rapa; Tha, Tarenaya hassleriana; Mdo, Malus domestica; Atr, Amborella trichopoda; Sit, Setaria italic; Jcu, Jatropha curcas; respectively. Overall sequence identity between Arabidopsis thaliana TDR and the TDR of Camelina sativa, Brassica rapa, Tarenaya hassleriana, Malus domestica, Amborella trichopoda, Setaria italic, Jatropha curcas is 89%, 84%, 78%, 60%, 56%, 50%, 46%, respectively. Residue numbers of A. thaliana TDR are indicated on the top the sequences. Each LRR repeat is indicated on the top of the sequences. The conserved TDIF interacting residues of TDR are shown in red, and the cysteine resides which form disulfide bonds in the TDR structures are depicted in dark yellow, and the five observed N-glycosylation site asparagine residues are colored in blue. The consensus residues are shown below each alignment. (TIF) [file pone.0175317.s008.tif]

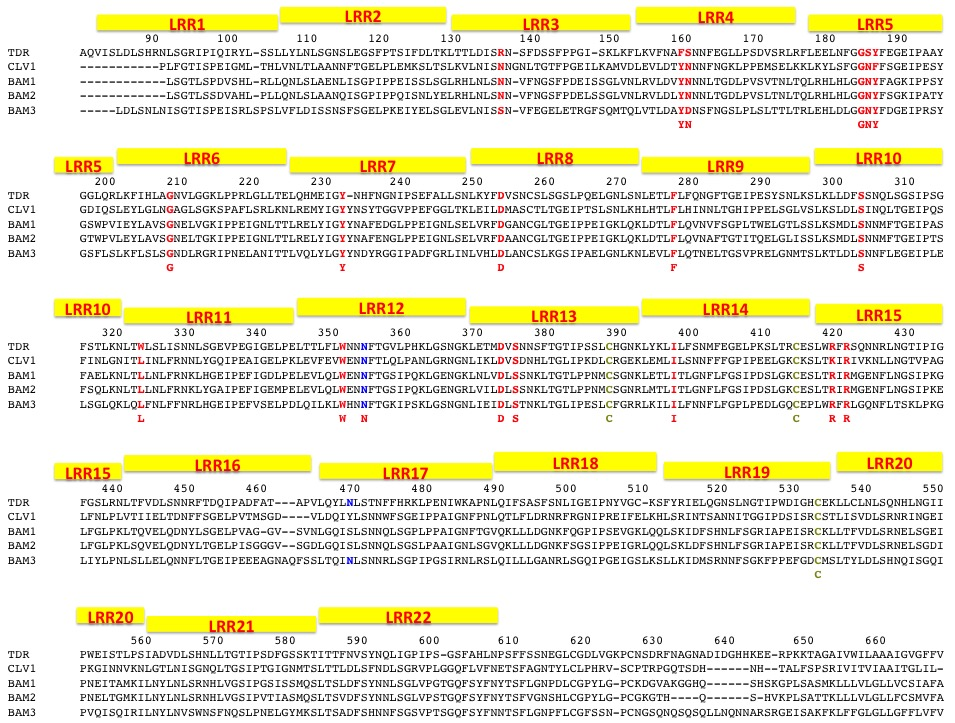

Supplement: S9 Fig — Residue numbers of A. thaliana TDR are indicated on the top the sequences. Each LRR repeat is indicated on the top of the sequences. The conserved TDIF interacting residues of TDR are shown in red, and the conserved cysteine resides which form disulfide bonds in the TDR structure are depicted in dark yellow, and the conserved N-glycosylation site asparagine residues are colored in blue. The consensus residues are shown below each alignment. (TIF) [file pone.0175317.s009.tif]

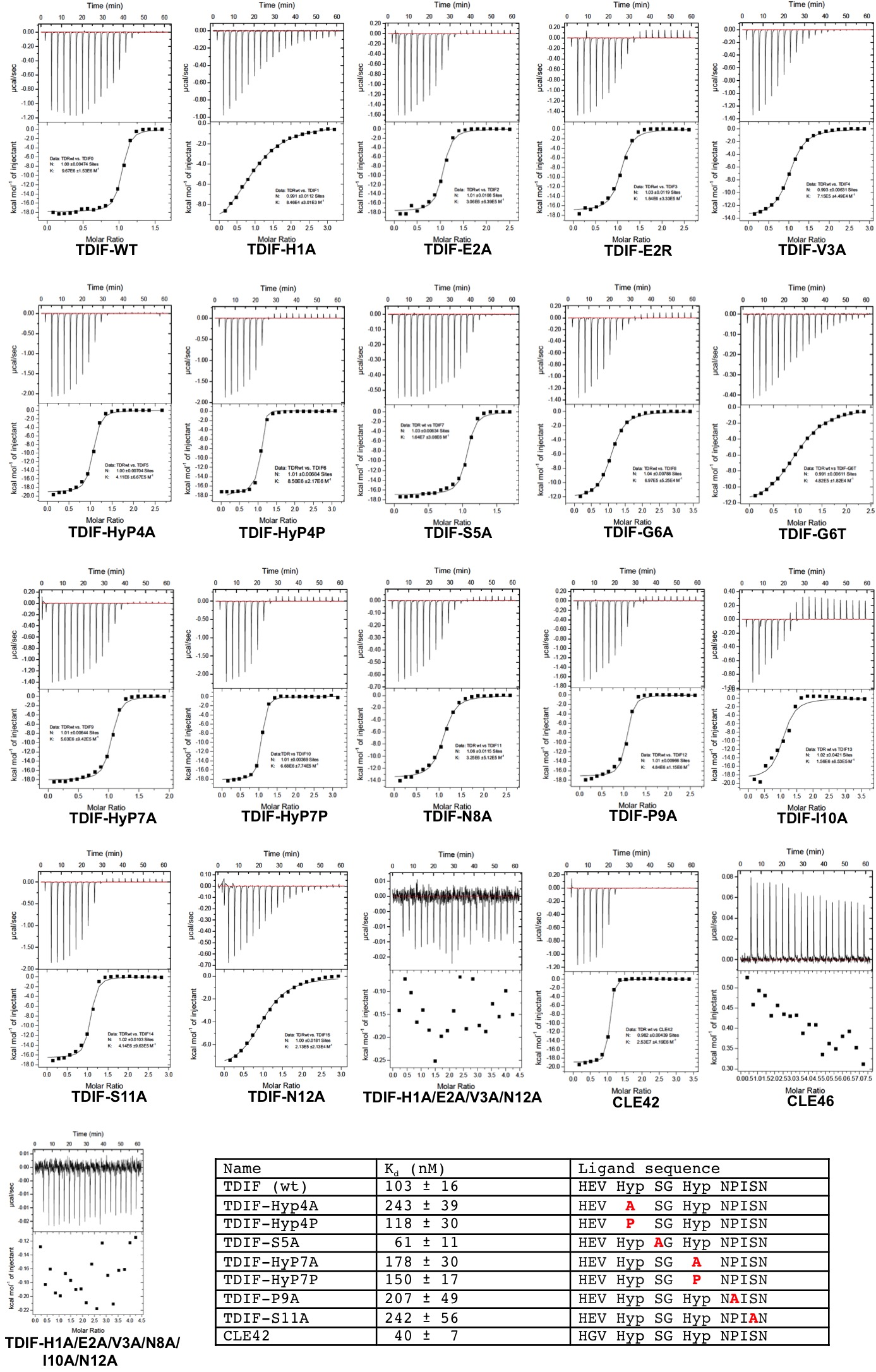

Supplement: S10 Fig — Kd values of the wtTDR with the TDIF mutants, CLE42 and CLE46 are shown along with their sequences in the table below. (TIF) [file pone.0175317.s010.tif]

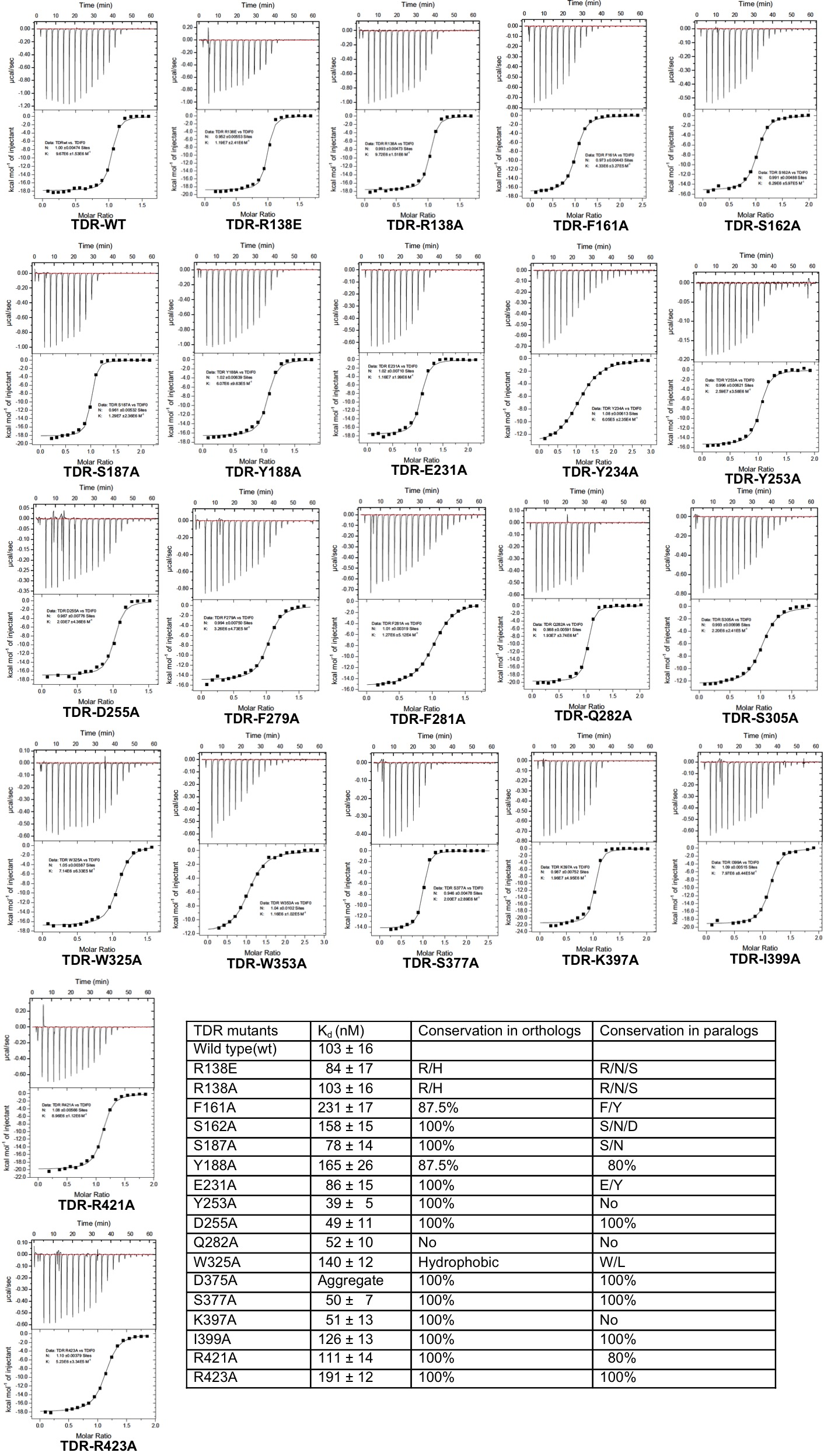

Supplement: S11 Fig — The measurements of the binding interactions (Kd values) between wtTDIF and ecdTDR mutants are shown in the table below. The sequence conservation of ecdTDR between orthologs and paralogs are also shown in the table. There are some residues that are not entirely conserved such as R138 which can be either R and H in orthologs and either R, N, S in paralogs. (TIF) [file pone.0175317.s011.tif]
